# Cooccurrence of Homologous Recombination Deficiency and Mismatch Repair Deficiency in Colorectal Cancer

**DOI:** 10.1002/mco2.70991

**Published:** 2026-09-15

**Authors:** Xu Zhang, Hao Xu, Kailai Wang, Wangxiong Hu

**Affiliations:** ^1^ Department of Ultrasound in Medicine The Second Affiliated Hospital of Zhejiang University School of Medicine Zhejiang University Hangzhou Zhejiang China; ^2^ Department of Clinical Laboratory Central Hospital of Jinhua City Jinhua Zhejiang China; ^3^ Cancer Institute (Key Laboratory of Cancer Prevention and Intervention National Ministry of Education) The Second Affiliated Hospital Zhejiang University School of Medicine Hangzhou Zhejiang China

**Keywords:** colorectal cancer, gene expression signature, homologous recombination deficiency, machine learning, mismatch repair deficiency

## Abstract

Homologous recombination deficiency (HRD) in colorectal cancer (CRC) remains largely unexplored. In contrast, mismatch repair deficiency (dMMR) occurs in ∼15% of patients with CRC. Although HRD and dMMR have historically been regarded as mutually exclusive, emerging evidence suggests that this mutual exclusivity may not be absolute. Here, we conducted a retrospective cohort study utilizing genomic and transcriptomic data to define HRD status in a Chinese dMMR CRC cohort (*n* = 99). Multiple machine learning approaches were employed to analyze the expression profiles of these tumors and to develop a classifier distinguishing HRD from homologous recombination proficiency (HRP) in dMMR CRCs. In the Chinese dMMR CRC cohort, 66% of tumors were classified as HRD. Compared with the HRP group, the HRD group had a significantly higher tumor mutational burden and better outcomes. The derived expression signature, comprising eight genes, successfully predicted HRD status in dMMR tumors with high accuracy in the training set (AUC = 0.88, Naïve Bayes) and the test set (AUC = 0.87). In this study, a subset of dMMR CRC tumors with co‐occurring HRD was identified, which may have potential implications for patient stratification and the application of targeted therapies, such as PARP inhibitors, in this molecular subgroup.

## Graphical abstract

Cooccurrence of dMMR and HRD in colorectal cancer. On the left blue shaded area, the dMMR (deficient mismatch repair) pathway is shown as a result of the loss of key DNA repair genes *MLH1*, *MSH2*, *MSH6*, and *PMS2*, which cause single strand breaks. On the right orange shaded area, the HRD (homologous recombination deficiency) pathway is presented, resulting from mutation of key genes *BRCA1*, *BRCA2*, ATM, and *RAD51* that lead to double strand breaks.

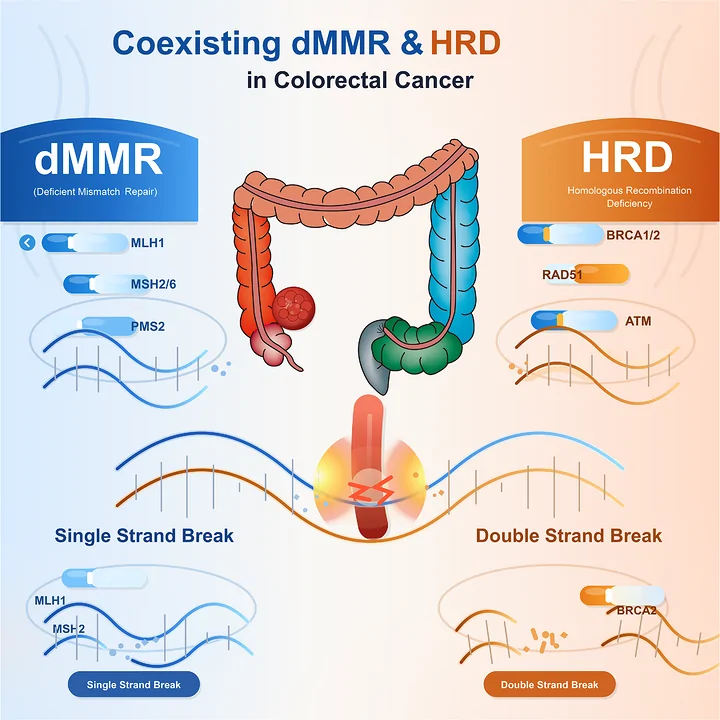

## 1 Introduction

Colorectal cancer (CRC) is a leading cause of cancer‐related mortality worldwide, and its pronounced molecular heterogeneity strongly influences therapeutic responses and clinical outcomes [1]. Two critical biomarkers, homologous recombination deficiency (HRD) and mismatch repair deficiency (dMMR), have emerged as important predictors of response to DNA‐damaging agents and immunotherapy, respectively [2, 3, 4, 5]. dMMR, which arises from defective single‐strand break repair and is often assessed by microsatellite instability‐high status, results in a hypermutated phenotype and is a well‐established biomarker for immune checkpoint inhibitor (ICI) therapy in CRC [6]. Conversely, HRD, which involves impaired double‐strand break repair, confers sensitivity to poly (ADP‐ribose) polymerase inhibitors and platinum‐based chemotherapy but has been rarely studied in CRC.

Historically, HRD and dMMR have been considered mutually exclusive in cancer [7, 8]. dMMR is relatively prevalent in CRC and gastric cancer, whereas HRD tends to occur more frequently in breast cancer (BRCA), ovarian cancer (OV), and pancreatic cancer [9]. This perceived exclusivity is largely grounded in the distinct etiologies of these deficiencies; dMMR is frequently driven by epigenetic silencing or detrimental mutations in *MLH1*, *MSH2*, *MSH6*, and *PMS2*, whereas HRD is commonly associated with the loss of BRCA1/2 and other double‐strand DNA break repair related genes such as *MRE11* and *RAD51* [10]. The findings of early genomic studies seemed to support this dichotomy, as dMMR tumors rarely exhibited the genomic scarring patterns [e.g., large‐scale state transitions (LST), telomeric allelic imbalance (TAI), and loss of heterozygosity (LOH)] characteristic of HRD and vice versa. This biological exclusivity has practical implications, as it traditionally guides a binary treatment decision‐making pathway: dMMR tumors toward ICI therapy and HRD tumors toward PARP inhibition [9].

However, emerging evidence suggests that this paradigm might be overly simplistic. With the advent of more sensitive genomic sequencing and advanced computational methods, rare cases of CRC exhibiting co‐occurring features of both HRD and dMMR have been documented, challenging the dogma of strict mutual exclusivity [9]. This development calls for more nuanced tools to accurately classify these complex molecular subtypes and to ensure that patients receive the most effective targeted therapies. Several studies have successfully employed machine learning (ML) to derive gene expression signatures (GESs) that predict HRD status in various cancer types, including BRCA and OV [11, 12, 13]. These signatures often outperform single‐gene biomarkers by capturing the complex transcriptional consequences of a deficient homologous recombination (HR) pathway.

The primary objective of this study was to leverage ML methodologies to develop and validate a novel GES capable of predicting HRD status specifically within the dMMR CRC tumors. By analyzing genomic and transcriptomic data from an assembled Chinese cohort, we aimed to test the historical assumption of mutual exclusivity and explore whether a subset of dMMR CRCs may also harbor an HRD phenotype, which could expand their therapeutic options. This research seeks to provide a refined molecular diagnostic tool that could better inform personalized treatment strategies for patients with intractable dMMR CRC.

## 2 Results

### 2.1 Prevalence of HRD in dMMR CRC

**FIGURE 1:**
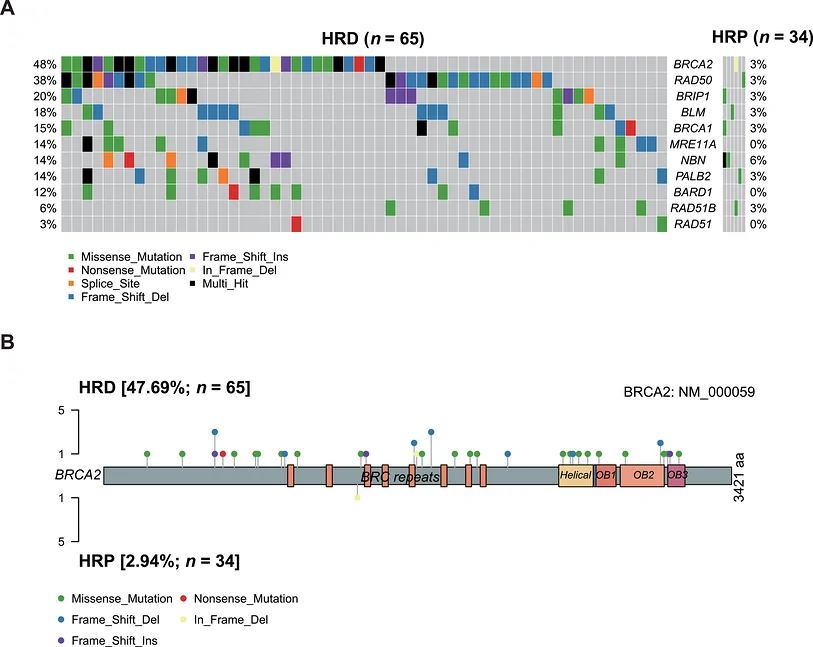
Mutational profile of the ZUCI dMMR CRCs. (A) Oncoplot of HRD‐related genes in the HRD and HRP groups. (B) Differences in the distribution of mutation sites in the *BRCA2* gene between the HRD and HRP groups. The oncoplots were generated by the *maftools* package (V2.28.0) in R.

A total of 99 Chinese patients aged 25–87 years (median age: 65 years) with confirmed dMMR CRC were included in the analysis; 43% were female. The disease was mainly at Stages II (57%) and III (26%); 43% had vascular invasion, and 20% had perineural invasion. Additionally, the tumor lesions were primarily located in the right colon (68%; Table 1), which was consistent with the findings of previous studies. Using the mutation‐defined HRD panel, a surprisingly high proportion of dMMR CRCs (*n* = 65, 66%) were classified as HRD in the Zhejiang University Cancer Institute (ZUCI) cohort (Figure 1A), with the remaining 34 samples defined as having homologous recombination proficiency (HRP). To verify this remarkably high HRD proportion in dMMR CRC, we conducted an independent validation using data from The Cancer Genome Atlas (TCGA) in parallel. Intriguingly, a similarly high frequency of HRD in dMMR CRC was also observed in the TCGA (41 out of 53, 77%) cohort, which ruled out potential racial bias. These findings confirm that a clinically relevant subset of dMMR tumors exhibit genomic scarring, which is indicative of HRD, despite the canonical understanding of mutual exclusivity between dMMR and HRD. In addition, we found that the mutation frequency of *BRCA2*, a key mediator of HR, was higher (48%) than that of other HR genes in the ZUCI cohort (Figure 1A,B).

**TABLE 1:** Differences in clinical characteristics between the HRD and HRP groups in this study.

Characteristics	Overall *n* = 99	HRD *n* = 65	HRP *n* = 34	*p* Value
Age	65 (51, 70)	63 (52, 70)	67 (51, 73)	0.5
Sex				0.2
Female	43 (43%)	31 (48%)	12 (35%)	
Male	56 (57%)	34 (52%)	22 (65%)	
Stage				0.7
I	9 (9.1%)	6 (9.2%)	3 (8.8%)	
II	57 (58%)	40 (62%)	17 (50%)	
III	26 (26%)	15 (23%)	11 (32%)	
IV	7 (7.1%)	4 (6.2%)	3 (8.8%)	
Vascular invasion	43 (43%)	26 (40%)	17 (50%)	0.3
Perineural invasion	20 (20%)	13 (20%)	7 (21%)	>0.9
Differentiation				0.7
I	28 (28%)	18 (28%)	10 (29%)	
II	67 (68%)	45 (69%)	22 (65%)	
III	4 (4.0%)	2 (3.1%)	2 (5.9%)	
Location				0.2
Left‐sided colon/rectum	32 (32%)	18 (28%)	14 (41%)	
Right‐sided colon	67 (68%)	47 (72%)	20 (59%)	

### 2.2 Correlation With Genomic and Clinical Features

**FIGURE 2:**
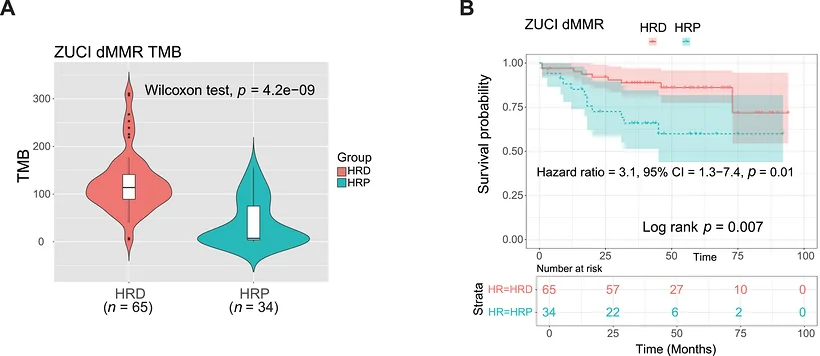
Differences in genomic and clinical outcomes between the ZUCI HRD and HRP groups. (A) Differences in TMB between the HRD and HRP groups. (B) Differences in OS between the HRD and HRP groups. Survival differences between the HRD and HRP groups were tested by the Kaplan–Meier method and analyzed with the log‐rank test with the functions *survfit* and *survdiff* in the *survival* package (V3.8‐3) in R.

We first investigated clinicopathological characteristics such as age, sex, TNM (tumor node metastasis) stage, lesion anatomical location, perineural and vascular invasion, and degree of differentiation and found that there were no significant differences between the HRD and HRP groups (all *p* > 0.05; chi‐squared test; Table 1). We further assessed the association between dMMR‐HRD status and other genomic metrics. Notably, compared with the HRP group, the HRD group had a significantly higher tumor mutational burden (TMB; mean 122 vs. 38, *p* = 4.2e−09; Figure 2A), confirming that concurrent deficiency in single‐strand break repair and double‐strand break repair exacerbated the underlying genomic instability.

**FIGURE 3:**
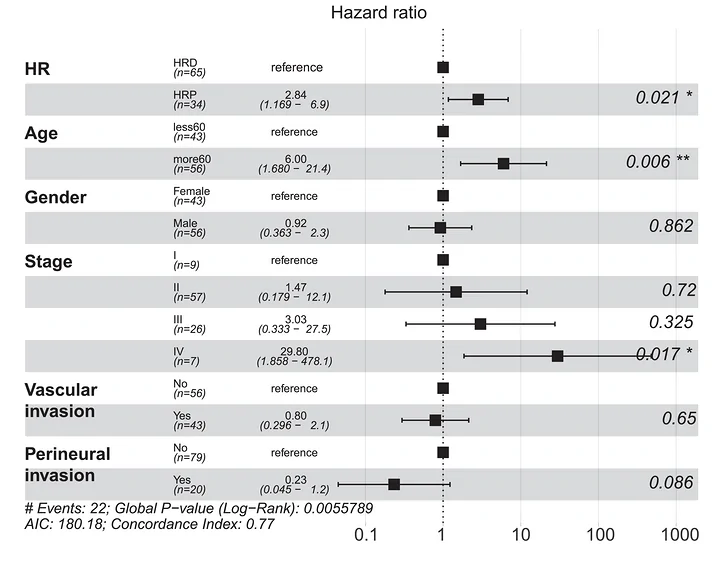
Multivariable Cox proportional hazard model and forest plot for dMMR CRC. The multivariable Cox analysis was performed with the *coxph* function in the R package *survival* and visualized by *survminer* package (V0.5.0) in R. *p < 0.05; **p < 0.01.

Given that there were no significant differences in the basic clinical characteristics between the HRD group and the HRP group but that the HRD group had a higher TMB, we conducted an exploratory analysis to investigate the differences in survival between the two groups. The results revealed that patients with dMMR CRC in the HRD group had better overall survival (OS) than those in the HRP group (*p* = 0.007, log‐rank test; Figure 2B). In this exploratory univariate Cox regression analysis, the risk was three times higher in the HRP group than in the HRD group (hazard ratio = 3.1; 95% confidence interval [CI]: 1.3–7.4; *p* = 0.01). The 5‐year survival rates were 86% (95% CI: 77–96%) and 60% (95% CI: 44–82%) in the HRD and HRP groups, respectively. Exploratory multivariate Cox regression further confirmed that the risk was significantly greater in the HRP group than in the HRD group (hazard ratio = 2.84; 95% CI: 1.2–6.9; *p* = 0.02) after adjusting for confounding factors such as age, sex, stage, and vascular and perineural invasion statuses (Figure 3); however, these results require validation in larger, prospective cohorts.

### 2.3 Development of a GES for dMMR CRC HRD Prediction

**FIGURE 4:**
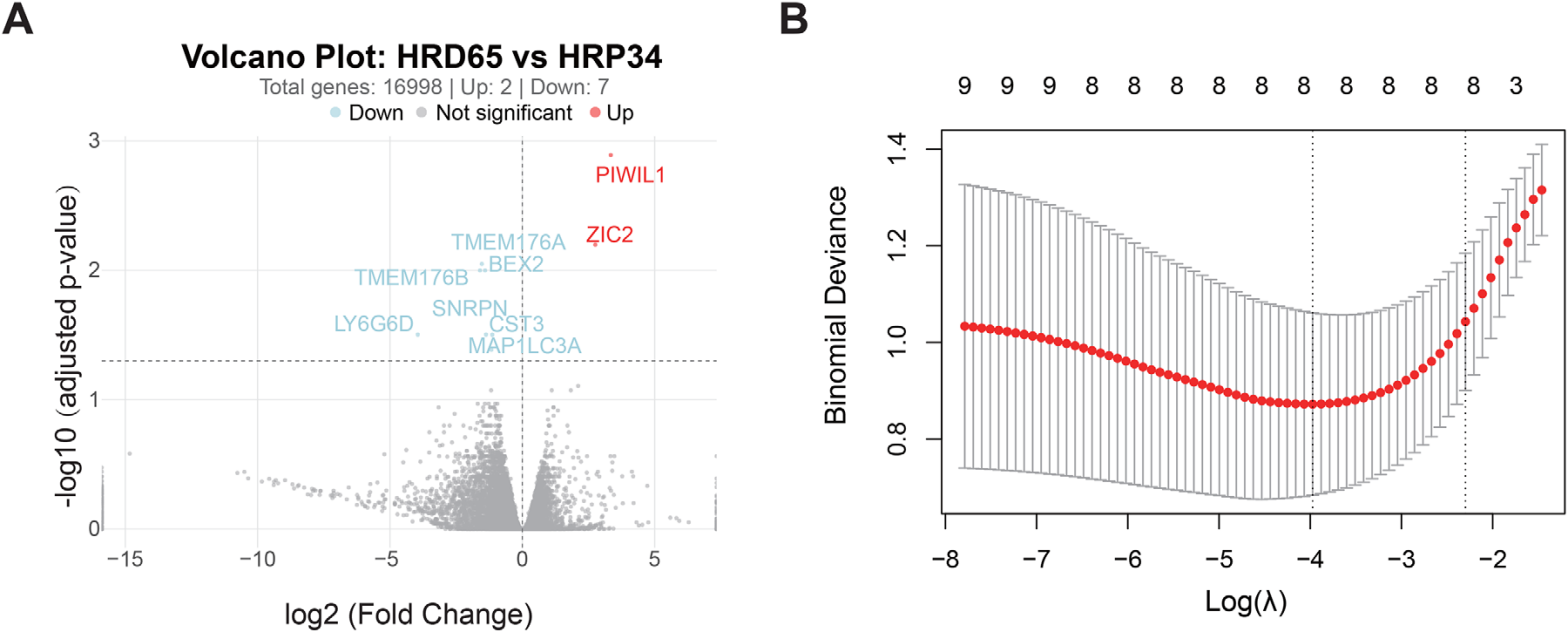
Feature selection process for model construction. (A) Volcano plot of differentially expressed genes between the HRD and HRP groups. (B) Feature selection performed by LASSO. The left vertical dotted line indicates where the CV error curve reached its minimum (lambda.min), and the right vertical dotted line indicates the most regularized model with a CV error within 1 standard deviation of the minimum (lambda.1se).

To identify a GES predictive of HRD status, we first performed differential gene expression analysis between the HRD and HRP groups. By comparing HRD (*n* = 65) tumors to HRP (*n* = 34) tumors, we identified nine significantly differentially expressed genes (adjusted *p* value < 0.05; absolute log2 fold‐change > 1; Figure 4A).

Using least absolute shrinkage and selection operator (LASSO) logistic regression with 10‐fold cross‐validation on the training set, we minimized overfitting and selected the eight most predictive genes (Figure 4B), including PIWIL1 (coefficient: −0.0248689065), ZIC2 (−0.0466788703), TMEM176A (0.0013319233), BEX2 (0.0097273905), TMEM176B (0.0014665094), LY6G6D (0.0123249217), SNRPN (0.0210196941), and CST3 (0.0003744754). The final signature, termed the dMMR‐HRD GES, was determined as a linear combination of the normalized expression values of these eight genes, weighted by their respective coefficients from the LASSO model.

### 2.4 Performance of the dMMR‐HRD Signature

**FIGURE 5:**
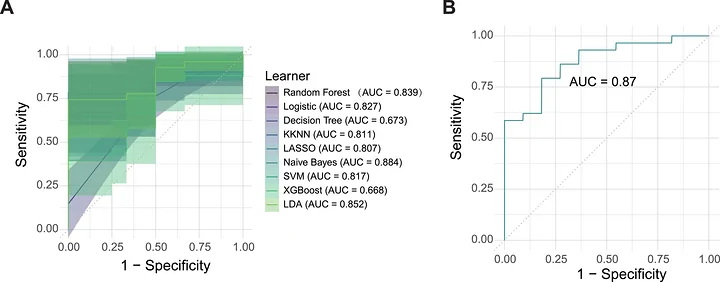
Model performance for discriminating HRD from HRP in dMMR CRC. (A) ROC curves for each ML model in the training ZUCI cohort. The Naïve Bayes model exhibited the best predictive performance. (B) ROC curve for the Naïve Bayes model in the test set.

The Chinese dMMR CRC cohort was randomly split into a training set (*n* = 69, 70%) and an independent validation set (*n* = 30, 30%). Clinicopathological characteristics were well balanced between the training and validation sets (all *p* > 0.05, chi‐squared test). The predictive performance of the dMMR‐HRD GES was first evaluated on the training set. We then employed nine ML classifiers to assess and optimize the performance of the signature. The signature revealed high discriminatory power, with an area under the receiver operating characteristic curve (AUC) ranging from 0.67 (XGBoost) to 0.88 (Naive Bayes, NB) for distinguishing HRD tumors from HRP tumors (Figure 5A). This performance was robustly validated in the held‐out independent test set (AUC = 0.87, NB; Figures 5B and S1). At the optimal cutoff point determined using the Youden index, the signature had a sensitivity of 81.8% and a specificity of 86.3% in the validation cohort.

## 3 Discussion

The identification of HRD has become increasingly critical in oncology, primarily because of its implications for sensitivity to PARPi therapy and platinum‐based chemotherapies [14]. In CRC, however, studies on HRD remain relatively scarce, partly because the perceived mutual exclusivity is rooted in the fundamental biological principle that a cell typically relies on one major DNA repair pathway at a time; the co‐occurrence of deficiencies in both pathways is thought to be biologically counterintuitive. Another reason might be that there is no unified definition standard similar to that for HRD in OV (i.e., score > 42) for CRC because the overall HRD score for CRC (median score = 10) is significantly lower than that for OV (median score = 46) [15, 16]. Therefore, in this study, we defined HRD according to a prespecified strict mutation threshold, namely, highly pathogenic mutations, including only frame shift insertion–deletion (INDEL) mutations and nonsense mutations in HR genes. Notably, our study challenges this dogma by revealing that a considerable subset of dMMR CRCs possesses a concurrent HRD phenotype, and the mutation‐defined HRD proportion would increase to 88% if missense mutations in HR genes were included for HRD determination. Rempel et al. [16] reported a negative correlation between the TMB and HRDsum in CRC, which may have been influenced by the calculation method (HRDscar derived from triple‐negative BRCA) of HRD scores. Furthermore, chromosomal instability is the main genomic characteristic of BRCA and OV; in contrast, LOH/LST/TAI and large‐scale copy number variation are less likely to be present in dMMR CRC, which is characterized by abundant point mutations and small INDELs. Thus, the HRD scoring system based on genomic scars has obvious limitations relating to its specificity to certain cancer types and is not fully applicable to CRC, especially dMMR CRC. In this study, the tailored ML model successfully identified a distinct 8‐GES that could predict HRD status in our cohort of dMMR CRCs. The performance metrics, including high AUC, sensitivity, and specificity, indicate that transcriptional profiling can serve as a promising tool for this purpose.

One important implication of our findings is the possibility of expanding the number of CRC patients who may benefit from PARPi therapy. This observation highlights key molecular concordance in dMMR CRCs across ethnicities (Asian and Caucasian patients with CRC) and emphasizes the value of genomic data for identifying shared targets, advancing precision oncology. By accurately identifying HRD within the dMMR subgroup, we can stratify patients who may derive dual or synergistic benefits from combined immunotherapy (targeting dMMR) and PARPi therapy (targeting HRD). This approach could provide new opportunities for improving treatment strategies in a disease where therapeutic options, especially in the metastatic setting, remain limited. Furthermore, the use of a gene expression‐based assay is highly compatible with routine clinical workflows, as it can be performed on standard formalin‐fixed paraffin‐embedded tissue samples, facilitating easier translation into diagnostic practice. Several genes included in our dMMR‐HRD signature may be mechanistically related to the HR pathway. For example, PIWIL1 may regulate the DNA damage response [17] and localize to the centrosome during mitosis, with its knockdown resulting in G2/M arrest and aberrant mitotic events in CRC cells [18]. Yan et al. [19] reported that TMEM176B diminishes DNA replication through regulation of the Wnt/β‐catenin signaling pathway. Moreover, Li et al. [20] found that methylation of *TMEM176A* acts as a synthetic lethal marker for ATM inhibitors in lung cancer through the ERK signaling pathway, which has been defined as a positive regulator of HR, and that HR is the most important DDR pathway in the late S and G2/M phases of the cell cycle. ZIC2 may also be involved in DNA replication through interactions with ZFP281 and BRCA2 [21, 22]. Together, these results provide a coherent biological basis for the model's reliability and interpretability.

Our results align with a growing body of evidence suggesting that the relationship between DNA repair pathways is more complex than the previously believed mutual exclusivity [23]. While the two deficiencies may be antagonistic from an evolutionary perspective, the genomic instability inherent in dMMR cancers could predispose them to acquiring secondary alterations that impair HR. This finding is important because the tumor microecosystem may not always be fully captured by its genetic drivers alone, such as *BRCA1*/*2* mutations. Understanding the complex interplay between tumor cell‐intrinsic mediators of disease progression is critical for the rational development of effective anticancer treatments [24].

This study has several limitations. Its retrospective nature and lack of direct evidence of platinum‐based treatment in dMMR CRC introduce potential selection biases. Although the sample size was sufficient for ML model development, validation in larger, multicenter prospective cohorts is required to confirm generalizability. Furthermore, the gold standard for defining HRD status in CRC remains debatable; we used a rigorous measure, but a universal definition for HRD in dMMR cancers is still needed. The most important next step is to correlate the HRD signature identified by our model with clinical response to PARPi therapy in patients with dMMR CRC within a prospective clinical trial. Additionally, exploring the mechanism underlying the cooccurrence of these deficiencies through in vitro and in vivo experiments is essential for understanding the biological mechanisms driving this phenomenon.

In conclusion, our ML‐derived GES effectively predicts mutation‐defined HRD in a considerable subset of dMMR CRCs, challenging the long‐held notion of strict mutual exclusivity between HRD and dMMR. This signature provides a practical tool for patient stratification and opens a promising new avenue for targeted therapy, suggesting a more nuanced, molecularly guided approach to treating this highly heterogeneous disease.

## 4 Materials and Methods

### 4.1 Study Design and Patient Cohort

This study was designed as a retrospective cohort analysis of freshly frozen tumor tissues and matched clinical data from patients diagnosed with dMMR CRC [25]. The Institutional Review Boards of all participating centers approved the study, and informed consent was waived given the retrospective nature of the work.

Eligibility required confirmation of dMMR status through immunohistochemical (IHC) detection of the loss of at least one core mismatch repair protein (MLH1, PMS2, MSH2, or MSH6). Enrollment spanned from January 2013 to December 2020 at ZUCI. Three pathologists specializing in CRC at the Second Affiliated Hospital, Zhejiang University School of Medicine, independently performed the IHC assessments in a blinded fashion within the pathology department. The Institutional Ethics Committee of the Second Affiliated Hospital of Zhejiang University, School of Medicine, granted ethical approval for this study (No. 2020‐666; Zhejiang, China). Patients were excluded if they (1) had missing or incomplete clinical data; (2) lacked fresh‐frozen tumor tissue samples and tumor‐matched normal adjacent tissues; or (3) had insufficient tumor tissue for DNA and RNA extraction or inadequate sequencing quality.

### 4.2 Whole‐Exome Sequencing and HRD Definition

DNA was extracted from the samples using a QIAamp DNA Mini Kit (QIAGEN). Library preparation followed the SureSelect XT Human All Exon V6 kit protocol (Agilent Technologies). Whole‐exome sequencing (WES) was carried out at the BGI Shenzhen, MGI R&D facility, with base calling performed via DNBSEQ. Raw sequencing data were subjected to quality control using SOAPnuke [26], during which reads containing adapter sequences, reads with unknown base N content greater than 10%, and low‐quality reads (defined as reads in which bases with a quality value below 10 accounted for more than 30% of the total bases) were filtered out. Clean reads were subsequently aligned to the human reference genome (hg38) using the Burrows‐Wheeler Aligner [27] with default parameters.

HRD status was assigned on the basis of a validated driver mutation gene panel. Consistent with a previously described approach [28], a tumor was classified as HRD‐positive if it harbored highly pathogenic mutations, specifically, frame shift INDEL mutations and nonsense mutations‐in HR genes, based on a prespecified strict threshold.

### 4.3 RNA Sequencing and Data Processing

*RNA Extraction and Sequencing*: Total RNA was isolated from freshly frozen tissue samples with a minimum tumor content of 20%. After RNA integrity was verified, sequencing libraries were prepared using the Illumina TruSeq RNA Access Library Prep Kit. Sequencing was performed on an Illumina HiSeq 4000 platform, targeting a depth of 50 million 150‐bp paired‐end reads.

*RNA‐Seq Data Processing*: Raw sequencing reads in FASTQ format were first evaluated for quality using SOAPnuke [29]. Adapter trimming, removal of low‐quality bases, and alignment of cleaned reads to the human reference genome (GRCh38) were conducted following the pipeline established in our previous work [25]. Gene‐level counts were generated using featureCounts from the Subread package. The expression level of each gene was quantified using RSEM (V1.3.1) [30].

### 4.4 Publicly Available Dataset Collection

Somatic mutational profiles and corresponding clinical information of patients with CRC from TCGA [31] were downloaded from the project's data portal (https://portal.gdc.cancer.gov/) on June 5, 2023. The HRD classification criteria applied to the TCGA samples mirrored those used for the ZUCI cohort and served exclusively to corroborate the observed HRD frequency.

### 4.5 ML‐Based Model Development

The cohort was randomly divided into a training set (70% of samples) and an independent hold‐out test set (30%). The training set was employed for feature selection and model building, whereas the test set was reserved solely for evaluating the performance of the final, locked model.

*Feature Selection*: To identify a GES predictive of HRD status, we first performed differential expression analysis between HRD and HRP tumors within the dMMR CRC cohort. This step was intended to isolate an HRD‐specific transcriptional signal distinct from the broader dMMR expression background. Analysis was performed using the *limma* package, and genes with an adjusted *p* value < 0.05 and an absolute log2‐fold‐change > 1 were retained as candidate features. Then, the candidate features that correlated with the dichotomy (HRD or HRP) were subjected to LASSO method in the *glmnet* package (V4.1‐8) to shrink the number of variables.

*Model Training and Validation*: We employed nine ML algorithms [LASSO, linear discriminant analysis, extreme gradient boosting (XGBoost), logistic regression (Logistic), random forest, KK‐nearest neighbors, support vector machine, decision tree, and NB] to predict the binary outcome (HRD vs. HRP). Regularization was incorporated to mitigate overfitting, and the hyperparameters for each model were optimized through 10‐fold cross‐validation on the training set. Model performance was quantified using AUC, accuracy, sensitivity, and specificity and visualized by *ggplot2* (V4.0.3) package.

### 4.6 Statistical Analysis

Unless otherwise specified, all the charts were drawn and all statistical computations were carried out in R (64‐bit, version 4.1.1). Categorical variables were compared using the chi‐squared test or Fisher's exact test, whereas continuous variables were analyzed using the Wilcoxon rank‐sum test. Statistical significance was set as a two‐sided *p* value of less than 0.05.

## Author Contributions

WXH designed the study. WXH performed TCGA and ZUCI analysis. WXH, HX, and XZ performed statistical analysis. XZ, KLW, and WXH wrote the manuscript. WXH reviewed and polished the manuscript. All authors read and approved the final manuscript.

## Funding

This work was supported by Fundamental Research Funds for the Central Universities (grant number: 226‐–2024‐00062) and National Natural Science Foundation of China (grant number: 81802883) to WXH and Social Development Projects of Jinhua Science and Technology Bureau (grant number: 2024‐3‐070) and Young and Middle‐aged Researchers Start‐up Fund of Jinhua Central Hospital (JY2023‐1‐01) to HX.

## Ethics Statement

This study was approved by the Institutional Ethics Committee of the Second Affiliated Hospital of Zhejiang University, School of Medicine (No. 2020‐666; Zhejiang, China). Given the retrospective nature of the study and the use of de‐identified patient data, the requirement for informed consent was waived by the ethics committee. The study was conducted in accordance with the ethical standards of the Declaration of Helsinki and its later amendments.

## Conflicts of Interest

The authors declare no conflicts of interest.

## Supporting information




**Supporting File**: mco270991‐sup‐0001‐SuppMat.docx.

## Data Availability

All data generated and/or analyzed during this study are referenced or included in this published article. The raw RNAseq and WES data were deposited in the National Genomics Data Center (https://ngdc.cncb.ac.cn/bioproject/) under project number (PRJCA021423).
