# Supplementary material for: Cooccurrence of Homologous Recombination Deficiency and Mismatch Repair Deficiency in Colorectal Cancer
Source: MedComm (2020). 2026 Sep 15;7(10):e70991. doi: 10.1002/mco2.70991 (PMC13575765; doi:10.1002/mco2.70991)
Supplement: Supplementary file 1 [file MCO2-7-e70991-s001.docx]

Cooccurrence of **homologous recombination deficiency** and **mismatch repair deficiency** in colorectal cancer

Running title: HRD and dMMR in CRC

Xu Zhang^1^, Hao Xu^2^, Kailai Wang^3^, Wangxiong Hu^3*^

**Affiliations:**

^1^ Department of Ultrasound in Medicine, The Second Affiliated Hospital, Zhejiang University School of Medicine, Hangzhou, Zhejiang, 310009, China

^2^ Department of Clinical Laboratory, Central Hospital of Jinhua City, Jinhua, Zhejiang, 321000, China

^3^ Cancer Institute (Key Laboratory of Cancer Prevention and Intervention, National Ministry of Education), The Second Affiliated Hospital, Zhejiang University School of Medicine, Hangzhou, Zhejiang, 310009, China.

*Correspondence to:

Wangxiong Hu

Tel: +86-571-87784606

Email: [wxhu@zju.edu.cn](mailto:wxhu@zju.edu.cn)

Figure S1

Fig S1. Flowchart for patient inclusion/exclusion and GES model development in this study.
